# Prospects and challenges of extracellular vesicle-based drug delivery system: considering cell source

**DOI:** 10.1080/10717544.2020.1748758

**Published:** 2020-04-08

**Authors:** Wanrong Meng, Chuanshi He, Yaying Hao, Linlin Wang, Ling Li, Guiquan Zhu

**Affiliations:** Department of Stomatology, Sichuan Cancer Hospital, Sichuan Key Laboratory of Radiation Oncology, School of Medicine, University of Electronic Science and Technology of China, Chengdu, PR China

**Keywords:** Drug delivery, extracellular vesicles, exosomes, microvesicles

## Abstract

Extracellular vesicles (EVs), including exosomes, microvesicles, and apoptotic bodies, are nanosized membrane vesicles derived from most cell types. Carrying diverse biomolecules from their parent cells, EVs are important mediators of intercellular communication and thus play significant roles in physiological and pathological processes. Owing to their natural biogenesis process, EVs are generated with high biocompatibility, enhanced stability, and limited immunogenicity, which provide multiple advantages as drug delivery systems (DDSs) over traditional synthetic delivery vehicles. EVs have been reported to be used for the delivery of siRNAs, miRNAs, protein, small molecule drugs, nanoparticles, and CRISPR/Cas9 in the treatment of various diseases. As a natural drug delivery vectors, EVs can penetrate into the tissues and be bioengineered to enhance the targetability. Although EVs’ characteristics make them ideal for drug delivery, EV-based drug delivery remains challenging, due to lack of standardized isolation and purification methods, limited drug loading efficiency, and insufficient clinical grade production. In this review, we summarized the current knowledge on the application of EVs as DDS from the perspective of different cell origin and weighted the advantages and bottlenecks of EV-based DDS.

## 1. Introduction

Extracellular vesicles (EVs), including exosomes, microvesicles (MVs), and apoptotic bodies, are nanosized membrane vesicles derived from most cell types. EVs are enriched in blood, saliva, and other biological fluids (Thery et al., 2002; Barile & Vassalli, 2017), carrying and delivering diverse biomolecules from their parent cells to receptor cells (Thakur et al., 2014; Thery, 2015). A growing body of evidence has shown that EVs are important mediators of intercellular communication and thus play significant roles in physiological and pathological processes, including stem cell maintenance, tissue repair, immune modulation, and tumor growth (Valadi et al., 2007; Thakur et al., 2014; Cheng et al., 2018a; He et al., 2018). As ‘molecule carrier’, EVs may serve as novel tools for various therapeutic and diagnostic purpose (EL Andaloussi et al., 2013; Ohno et al., 2016), such as anti-tumor therapy (Poggio et al., 2019), immune-modulatory (Buzas et al., 2014), and drug delivery (Gudbergsson et al., 2019). As a drug delivery vesicle, EVs have been tested for the delivery of siRNAs (El-Andaloussi et al., 2012), miRNAs (Li et al., 2019c), proteins (Haney et al., 2015), small molecule drugs (Zhuang et al., 2011), nanoparticles (Jung et al., 2018), and CRISPR/Cas9 (Lin et al., 2018) into animal models. Owing to their natural origin, EVs are born with high biocompatibility, enhanced stability, and limited immunogenicity, which provide potential advantages over traditional synthetic delivery vehicles, such as liposomes and nanoparticles.

Liposomes are round bubbles consisting of an aqueous core encapsulated by natural or synthetic phospholipids (Fan & Zhang, 2013). With this structure, liposomes are ideal drug carriers since hydrophilic drugs tend to be entrapped in the core, while hydrophobic ones will be entrapped within the lipid bilayers (Gulati et al., 1998). Liposomes have been an important choice for drug since their discovery by Alec Bangham and colleagues in 1961 (Bangham & Horne, 1964). Indeed, liposomes are particularly potent in the treatment of some types of cancer, in which they can achieve passive targeting via the leaky tumor vasculature, according to the enhanced permeability and retention (EPR) effect (Torchilin, 2005). As a result of all the advantages of liposomes as drug delivery systems (DDSs), several liposomal drug products are currently available in the market, such as Doxil^®^, Ambisome^®^, DaunoXome^®^, Marqibo^®^, Onivyde™, Myocet^®^, etc. (Antimisiaris et al., 2018), while others are under clinical testing, such as CPX-1, CPX-351, En-doTAG-1 (Lip-opack), LEP-ETU, etc. (Palazzolo et al., 2018). Products like Doxil^®^ where the main pharmacological mechanism is passive targeting have poor selectivity toward cancer cells, which induce severe systemic side effects (Antimisiaris et al., 2018). To obtain active target ability in diseased sites, surface ligand modification of liposomes is mostly used in the literature (Jiang et al., 2019). However, clinical trials of these modified liposomes turned out to be barely effective (Allen & Cullis, 2013). The reasons turned out to be that (1) an anatomical barrier exists within tumor; (2) the targeted ligands are unstable and inactive on the membrane surface; and (3) the ligand is insufficient to trigger the binding of the target (Nogueira et al., 2015; Juliano, 2016). Fortunately, EVs have great potential for natural drug delivery, because they can penetrate through anatomical barriers (Rufino-Ramos et al., 2017; Das et al., 2019), keep stability (Zhang et al., 2018a), and maintain sufficient binding effects (Tian et al., 2018). Although EVs’ merits make them ideal for drug delivery, EV-based drug delivery remains challenging, such as short of standardized isolation methods, low drug loading efficiency, and restricted clinical grade production. More importantly, no consensus has been gained on the cell type that could serve as an ideal source of drug delivery-grade EVs. Since EVs inherit parent cell features, EVs derived from different cell types may vary in delivering and targeting properties.

In this review, we summarized the current knowledge on the application of EVs as DDSs from the perspective of different cell origin.

## 2. Biochemical properties of EVs

**Figure 1.**
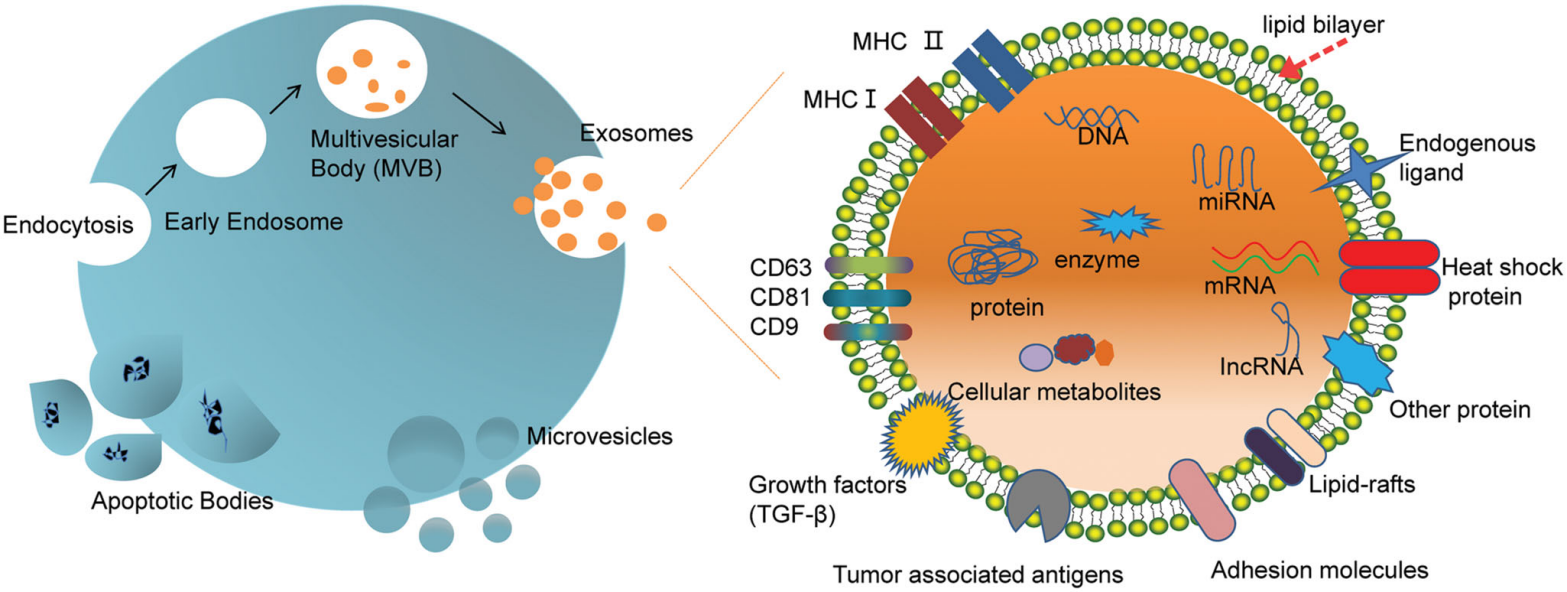
Scheme of biogenesis of three types of extracellular vesicles (exosomes, microvesicles, and apoptotic bodies) and component of exosome. Exosomes are cell secreted vesicles of ∼100 nm in size and packed with a variety of cellular components including mRNAs, miRNAs, proteins, enzymes, lipids, carbohydrates, etc. The exosome surface is decorated with various membrane proteins responsible for different pathophysiological functions.

Based on the size and biogenesis process, EVs can be divided into three main subpopulations: (i) exosomes, (ii) microvesicles, and (iii) apoptotic bodies. Here, we focus on the first two classes of EVs, which have potentials in drug delivery. Exosomes and MVs are two different forms of EVs representing generic terms for diverse, nanoscale membrane vesicles released by cells (Tkach & Théry, 2016) (Figure 1).

### 2.1. Biogenesis of EVs

EVs were initially illustrated in 1983 when two independent groups found that multivesicular bodies in reticulocytes released such vesicles into the extracellular environment (Pan & Johnstone, 1983; Harding et al., 1984). Few years later, several other groups reported that exosomes are formed by inward budding of limiting membrane of endosomes (Gruenberg & Maxfield, 1995; Babst et al., 2000). Thus, the cytosolic proteins, such as heat shock cognate 70 (HSC70), annexin II, major histocompatibility complex class II (MHC-II), and CD63 (Thery et al., 1999), are found to express on the membrane of exosomes. In contrast, MVs and apoptotic bodies are generated by the outward budding and fission of the cell membrane (EL Andaloussi et al., 2013). Apoptotic bodies are typical membrane blebs released by cells undergoing apoptosis in a wide range of 50–5000 nm. These vesicles contain membrane-enveloped fragments of apoptotic DNA and cytoplasm. They carry ‘find-me’ and ‘eat-me’ molecular signals to attract phagocytes to apoptotic sites and promote clearance of apoptotic cells (Poon et al., 2014). Microvesicles, 20–1000 nm in diameter, are generated by budding from the plasma membrane (Cocucci et al., 2009). Owing to the biogenesis process, the membrane composition of MVs reflects their parent cell more closely than exosomes. Furthermore, owing to highly regulated biogenesis process, exosomes typically accommodate some additional defined components (Thery et al., 2009). As novel DDSs, both MVs and exosomes have their merits depending on specific purpose.

A growing body of data suggests that biogenesis of EVs is a very sophisticated regulation process governed by a set of signaling molecules, which is beyond the purpose of this review and has been discussed in several other reviews (Alderton, 2012; Ruivo et al., 2017; van Niel et al., 2018; Gurunathan et al., 2019). Since most of the literatures have disregarded the different origins of exosome and MV, it is unfeasible to identify which one is really counted. Thus, in this review, the term exosome and MV were used per reference indicated.

### 2.2. Composition and functions of EVs

Biochemical and proteomic analyses of EVs have revealed the presence of lipids, proteins, nucleic acids, and other components. Several databases (i.e. ExoCarta (Keerthikumar et al., 2016), Evpedia (Kim et al., 2015), and Vesiclepedia (Kalra et al., 2012)) have been built up to provide information on EV cargos. According to updated ExoCarta, 9769 proteins, 3408 mRNAs, 2838 microRNAs (miRNAs), and 1116 lipids have been identified in EVs. The composition and content of EVs vary from each other depending on their parent cells, physiological conditions, and environmental stimulation (Mignot et al., 2006; Clayton & Mason, 2009).

EVs play a pivotal role in intercellular communication by carrying biofunction molecules, such as proteins, mRNAs, and miRNAs. As cell–cell mediators, EVs not only regulate various normal physiological activities but also participate in initiation and progression of tumor. We have previously found that the hypoxic microenvironment may stimulate tumor cells to generate miR-21-rich exosomes that are delivered to normoxic cells to promote metastatic behaviors (Li et al., 2016). Moreover, hypoxic tumor cell-derived exosomes could deliver miR-21 to myeloid-derived suppressor cell (MDSC), inducing immunosuppressive ability through miR-21/PTEN/PD-L1 pathway (Li et al., 2019b).

Researchers indicated that EVs play dual roles in regulating tumor growth depending on the source of EVs (Gargiulo et al., 2019; Han et al., 2019; Vermeer, 2019). For example, EVs derived from mature dendritic cells (DCs) contain phospholipids comprised of C22:6 docosahexaenoic acid (DHA), which enhance the antigen presenting ability of DCs and thus inhibit tumor cell proliferation (Pitt et al., 2016). However, EVs derived from cancer cells carry tumorigenic miRNA which significantly promoted tumor cell proliferation (Li et al., 2016). Thus, to better meet clinic application of EV-based drug delivery, we have to take the source of EVs in to consideration, which will be discussed below.

### 2.3. Isolation and characterization of EVs

The most common procedure to purify EVs from cell culture supernatants involves a series of centrifugations to remove dead cells and large debris, followed by a final high-speed ultracentrifugation to pellet EVs (Thery et al., 2006). In addition to traditional isolation method, several methods have been developed to efficiently isolate EVs from cells and biological fluids: (1) ultracentrifugation-based isolation techniques; (2) size-based isolation techniques; (3) immunoaffinity capture-based techniques; (4) EVs precipitation; (5) microfluidics-based isolation techniques (Li et al., 2017). Of course, each method has its advantages and disadvantages, and the appropriate method of isolation should depend on the purpose of our research.

Although EVs are considered to be heterogeneous, they are expected to be universal when being applied as a DDS (Armstrong & Stevens, 2018). For both research and clinical purposes, several characterization and validation methods have been developed to analyze EVs purity and to quantify EV cargos. These methods include transmission electron microscopy (TEM), scanning electron microscopy (SEM), atomic force microscopy (AFM), nanoparticle tracking analysis (NTA), dynamic light scattering (DLS), resistive pulse sensing, enzyme-linked immunosorbent assay (ELISA), flow cytometry, fluorescence-activated cell sorting (FACS), and microfluidics and electrochemical biosensors (Dragovic et al., 2011; Hartjes et al., 2019).

## 3. EVs-based drug delivery systems

### 3.1. ‘Factories’ for EVs

**Figure 2.**
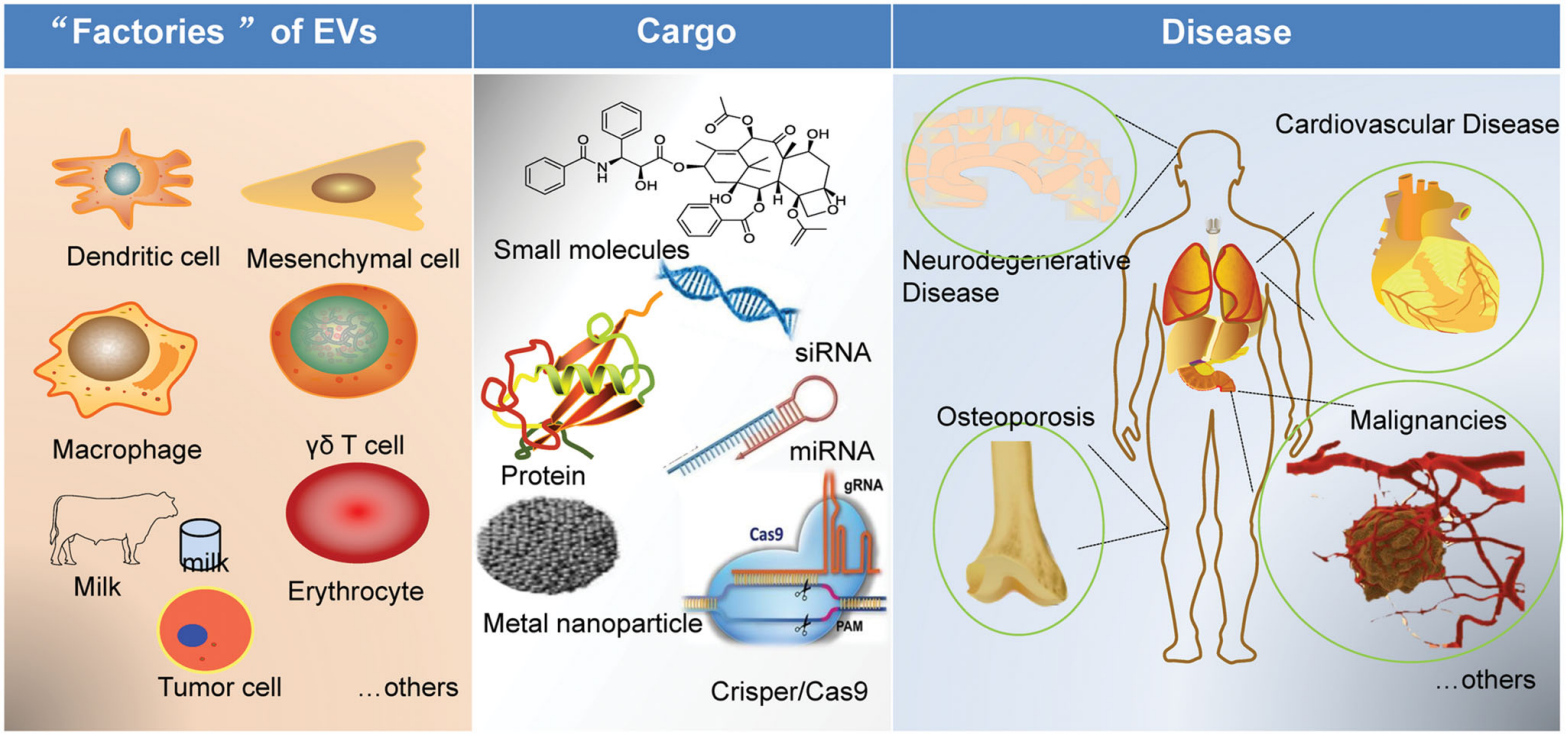
Scheme of the potential of EVs in disease treatment and drug delivery. EVs can be isolated from different ‘factories’ (dendritic cells, mesenchymal stem cells, macrophages, milk, tumor cells, others), loading different cargos (small molecules, nucleic acids, protein, metal nanoparticles), and targeting to precise disease (cardiovascular disease, neurodegenerative disease, osteoporosis, cancer, malignancies, and metastasis).

Although most cells can produce EVs, not all cell-derived EVs are suitable for drug carriers. Drug delivery-scale EVs should have strict quality standards, such as surface protein, size, yield, and intracavitary composition. Several types of cells have been investigated for their potential application as EV donators for drug delivery (Figure 2).

#### 3.1.1. Dendritic cells

As the antigen-presenting cells (APCs), DCs play a central role in initiating antigen-specific immunity and tolerance (Steinman, 2012). In cancer, DCs act as the initial link between oncogenesis and the host immune system, the first step of a cancer/immunity cycle that aims to eliminate cancer cells through the activation of T cells (Chen & Mellman, 2013). Immunogenic signals, such as proinflammatory cytokines, danger-associated molecular patterns (DAMPs), or pathogen-associated molecular patterns (PAMPs) could trigger DCs to present captured tumor-associated antigens (TAAs) via MHC-I and MHC-II molecules to T cells in cooperation with costimulatory molecules such as CD80 and CD86, resulting in priming and activation of TAA-specific effector T cells. However, such T cell responses can only be generated if certain additional conditions are met in the local environment (Chen & Mellman, 2013). DC-based immunotherapy is also challenging in clinical practice (Kantoff et al., 2010), because DCs are too large to penetrate deep into tumor and are difficult to store over long periods of time while maintaining their efficacy (Pitt et al., 2014).

The use of DC-derived EVs (DEVs) has been heralded as a solution to many technical challenges associated with DC-based immunotherapy, because DEVs maintain the essential immunostimulatory faculties of DCs (e.g. the ability to present antigens to T cells) and the stable nature of EVs allows their frozen storage for at least 6 months (Andre et al., 2004). DEVs are also more amenable to a strictly regulated and monitored manufacturing process (e.g. their composition and MHC-I and MHC-II content can be easily defined), and they lack the risks associated with viable cellular or viral therapies such as the risk of *in vivo* replication (Gabrilovich et al., 1996; Zhang et al., 2014a). In the last decade, DEV-based therapy was not only launched in immunotherapy but also applied in drug delivery.

The work initially described by Alvarez-Erviti et al. (2011) in 2011 demonstrated that DEVs can be developed for targeted RNA interference (RNAi) delivery to the brain after systemic injection using RVG-targeted exosomes. They provided the first proof-of-concept research for the potential of these naturally occurring vesicles for drug delivery. The next year, El-Andaloussi et al. (2012) provided a protocol that first described the generation of targeted exosomes through transfection of an expression vector. Next, they explained how to purify and characterize exosomes from transfected cell supernatant. Then, they detailed crucial steps for loading siRNA into exosomes and finally they outlined how to use exosomes to efficiently deliver siRNA *in vitro* and *in vivo*. In the following years, there are emerging number of works that associated with DEVs for the drug delivery (Lakhal & Wood, 2011; Cooper et al., 2014; Tian et al., 2014; Liu et al., 2017; Sabado et al., 2017; Pullan et al., 2019). Recently, EVs, such as DEVs are considered as a novel shuttle for delivery of therapeutics across biological barriers (Das et al., 2019). This novel EVs-based delivery approach holds biocompatibility and low immunogenicity *in vivo* (Pullan et al., 2019).

According to the multiple applications of DEVs, we can conclude that DEVs are candidates for immunotherapy and drug delivery (Lakhal & Wood, 2011; Pitt et al., 2016; Sabado et al., 2017). The antigen-presenting molecules, MHC-I, II, and T cell co-stimulators are enriched in DEVs (Pitt et al., 2016). Thus, for DDSs, DEVs will play dual effects of immunity and anti-tumor therapy in treatment of cancer (Pitt et al., 2016). Moreover, DEVs can overcome the biological barriers, such as blood–brain barrier (BBB), making them more attractive in future drug delivery (Khan et al., 2018).

#### 3.1.2. Mesenchymal stem cells

Mesenchymal stem cells (MSCs) and easily accessible primary cells can be harvested from a large variety of tissues (Lee et al., 2004; Kern et al., 2006), such as adipose tissue (Lee et al., 2004; Banas et al., 2007), umbilical cord blood (Kern et al., 2006), liver (Götherström et al., 2003), amniotic fluid (Roubelakis et al., 2007), and placenta (Miao et al., 2006) as well as dental pulp (Huang et al., 2009; Lai et al., 2010). These cells can differentiate into both mesenchymal and non-mesenchymal cells (Sato et al., 2011). The convenience of isolation and specialized biological functions of MSCs make them a popular choice for cell therapy in preclinical and clinical trials. Early in 2004, Nakamura et al. (2004) initially described the antitumor effect of engineered MSCs in a rat glioma model. Since then, there were emerging works that utilized MSCs in gene therapy and drug delivery (Kim et al., 2010; Sun et al., 2011; Hsiao et al., 2012; Lee et al., 2014; Choi et al., 2015). Hu et al. (2011) demonstrated that human umbilical blood mononuclear cell-derived MSCs serve as interleukin-21 gene delivery vehicles for epithelial ovarian cancer therapy in nude mice. Pessina et al. (2011) provided a new approach by MSCs primed with paclitaxel loading for cancer therapy loaded with paclitaxel. Thus, MSCs have been regarded as an ideal carrier for drug and gene delivery.

Recently, it is proposed that MSCs might exert their therapeutic effects mainly through secreted extracellular factors (Wen et al., 2016). As EVs are involved in cell–cell communication, it is hypothesized that EVs mediate the paracrine effects of MSCs (Mancuso et al., 2019). MSC-derived EVs (MEVs) have been revealed to have similar function of MSCs, such as facilitating the repair of kidney injury (Yao & Ricardo, 2016), modulating immune responses (Zhang et al., 2014b), promoting wound healing (Gregoire et al., 2015), and drug delivery (Lai et al., 2013).

Munoz et al. (2013) reported an increase of miR-9 in temozolomide (TMZ)-resistant glioblastoma multiforme (GBM) cells. They first delivered anti-miR-9 to resistant GBM cells with MEVs, resulting in a decreased expression of multidrug transporter and sensitization of the GBM cells to TMZ. From then on, MEVs have been increasingly considered as substitutes for MSCs in drug delivery. Efforts have been made to improve the efficacy of MEV-based drug delivery for clinical use (Cheng et al., 2018b; Lang et al., 2018; Sharif et al., 2018; Jo et al., 2019; Li et al., 2019a; Perets et al., 2019; Riazifar et al., 2019). For example, a good manufacturing practice (GMP) standard for large-scale clinical MEVs production based on bioreactor has been established (Mendt et al., 2018). Bone marrow MSCs (BM-MSCs) were developed to be candidates for the ‘factory’ of drug delivery EVs. Together, MEVs have added advantages in terms of easy expansion and harvesting and low immunogenicity (Munoz et al., 2013; Kourembanas, 2015; Haraszti et al. 2018; Phan et al., 2018), which enforces efficiency in the delivery of drugs.

#### 3.1.3. Macrophages

Macrophages are a group of heterogeneous monocyte–macrophage lineage cells which play critical roles in innate immunity and also initiate the adaptive immune response. Macrophages display diverse phenotypes in response to different stimuli, and they are categorized into M1 and M2 subsets (Cheng et al., 2017). Cheng et al. (2017) demonstrated that M1, rather than M2, macrophage-derived exosomes enhanced activity of lipid calcium phosphate nanoparticle-encapsulated Trp2 vaccine and induced a stronger antigen-specific cytotoxic T cell response. Moreover, EVs from macrophages are known to express functional immune modulating proteins including MHC class I and MHC class-II (Pitt et al., 2016), which preferentially induce Th1-type (cell-mediated) immune response that directs T cells to attack abnormal cells (such as cancer cells) or cells infected with intracellular parasites (Segura et al., 2005; Lynch et al., 2009; Yao et al., 2013).

The naïve macrophage (Mϕ) EVs can penetrate through the BBB in mammals. The mechanism might be that these EVs contain integrin lymphocyte function-associated antigen 1 (LFA-1), intercellular adhesion molecule 1 (ICAM-1) (Jin et al., 2018), and carbohydrate-binding C-type lectin receptors (Aranda-Souza et al., 2019), that can interact with brain vessel endothelial cells comprising the BBB (Yuan et al., 2017). Notably, upregulation of ICAM-1, a common process in inflammation, promoted Mϕ EVs’ uptake by BBB. By means of this, naïve Mϕ exosomes, after intravenous administration, could cross the BBB and deliver the brain-derived neurotrophic factor (BDNF), into brain (Yuan et al., 2017). Similarly, Haney et al. (2015) reported earlier that macrophage-derived exosomes loaded with catalase can be considerably detected in brain of mouse with Parkinson’s disease following intranasal administration. Kim et al. (2016) encapsulated low molecular chemotherapeutics, such as paclitaxel, to macrophage exosomes, resulting in an increased cytotoxicity more than 50 times in drug resistant cancer cells. They further developed and optimized the formation of macrophage-exosome with incorporated aminoethyl anisamide-polyethylene glycol (AA-PEG) vector moiety to target the sigma receptor, which is overexpressed by lung cancer cells. Polyethylene glycol (PEG) reduced the recognition and clearance by the mononuclear phagocyte system. To this end, Kooijmans et al. (2016) have recently shown that the introduction of PEG to exosomes resulted in stealth properties, which significantly increased their circulation time in mice. The AA-PEG modified exosomes loaded with PTX (AA-PEG-exoPTX) possessed a high loading capacity, profound ability to accumulate in cancer cells upon systemic administration, and improved therapeutic outcomes (Kim et al., 2018).

On the contrary, IL-4-activated macrophage-derived exosomes were found to deliver miR-223 to breast cancer cells, eliciting an invasion potential of recipient cancer cells (Yang et al., 2011). Additionally, macrophage-derived exosomes are suggested to transfer miR-365, a key regulator of gemcitabine resistance in pancreatic adenocarcinoma (Binenbaum et al., 2018). Hence, macrophages have been realized to engage in a yin-yang balance in cancer development with both tumor inhibiting and promoting roles. Thus, macrophage-centered therapeutic approaches, including macrophage EV-based approaches, need to overcome macrophage-sustained tumor promotion and take advantage of macrophage antitumor potential before entering the clinical arena (Mantovani et al., 2017).

#### 3.1.4. Milk

Bovine milk consumption is generally considered to be safe and provides important nutritional benefits (Haug et al., 2007). Inspired by this natural phenomenon, a large number of studies have developed oral formulations by using milk EVs as chemotherapeutic packages which protected from the low pH and degradative enzymes in the stomach (Admyre et al., 2007; Johnsen et al., 2014; Munagala et al., 2016).

Melnik et al. (2014) suggested that bovine milk exosomes provide a viable alternate with high impact because of cost effectiveness, biocompatibility, stability, tumor target ability, and lack of toxicity. Due to their known stability in acidic environment (Aqil et al., 2017), milk exosomes may provide additional benefit as a desirable oral drug delivery carrier, with wide therapeutic applications. Munagala et al. (2016) reported that raw mature bovine milk can serve as a biocompatible and cost-effective source for harvesting bulk quantities of exosomes and that milk exosomes have tremendous potential as a drug carrier for hydrophilic and lipophilic agents, including chemo drugs. Drug loaded in milk exosomes showed significantly higher efficacy compared with free drug against lung tumor xenografts *in vivo*. Paclitaxel-loaded milk exosomes delivered orally showed significant tumor growth inhibition against human lung tumor xenografts in nude mice compared with i.p. injection of paclitaxel (Agrawal et al., 2017). Moreover, paclitaxel-loaded milk exosomes showed remarkably lower systemic and immunologic toxicities compared with i.v. injection of paclitaxel. Betker et al. (2019) further demonstrated milk-derived exosomes are absorbed from the gastrointestinal tract via the ‘neonatal’ Fc receptor, keeping intact after absorption When encapsulated in milk exosomes, curcumin showed enhanced stability, solubility, and bioavailability (Vashisht et al., 2017). Milk-derived exosomes are also suggested to be a viable natural nano-carrier for siRNA delivery in therapeutic application against cancer (Aqil et al., 2019).

It is interesting to develop milk EVs for novel drug delivery due to the merits of cost effectiveness, biocompatibility, physical and biological stability, scalability of manufacturing process, versatility of agents it can carry, and ability to function with ligands for targeting (Munagala et al., 2016). However, further studies are needed to figure out any potential toxicity with long-term use of milk exosomes.

#### 3.1.5. Tumor cells

It is suggested that tumor cell-derived EVs (TEVs), especially autologous TEVs, carry tumor antigen repertoires, costimulatory molecules, and DNA fragments similar to their parental cells (Qiu et al., 2019; Rahbarghazi et al., 2019). This nature could elicit a potent T cell-dependent anti-tumor immune response and achieve therapeutic effects in mouse models of melanoma (Mannavola et al., 2019), hepatocellular carcinoma (Moris et al., 2017), and colon carcinoma (Teng et al., 2017). Compared with EVs produced by noncancerous cells, TEVs can achieve tumor cell-specific targeting by utilizing the intrinsic homo-adhesion characteristics mediated by the surface antigen of the membrane (Aslan et al., 2019).

In 2001, TEVs were initially uncovered as a novel source of tumor-rejection antigens for T-cell cross priming (Wolfers et al., 2001). Thereafter, much knowledge has been gained on role of TEVs in tumorigenesis but only very recently they were used in DC-based immunotherapy (Mahaweni et al., 2013). It is shown that mice received TEVs-educated DC immunotherapy had an increased survival compared to those received tumor lysate-loaded DCs (Mahaweni et al., 2013). Recently, Bose et al. (2018) investigated a TEVs-based nanoplatform for miRNA delivery as well as the magnetic resonance imaging (MRI) of cancer. They demonstrated that the distribution of TEVs correlated well with the tumor-targeting capability doxorubicin combination treatments. More recently, Guo et al. (2019) conducted a human study of intrapleural delivery of a single dose of autologous tumor cell-derived microparticles packaging methotrexate (ATMPs-MTX), showing that manufacturing and infusing ATMPs-MTX were feasible and safe, without evidence of toxic effects of grade 3 or higher. Besides, notable reductions in tumor and CD163+ macrophages in malignant pleural effusion after ATMP-MTX infusion were observed (Guo et al., 2019). These promising results suggest that autologous TEVs packaging molecules and nanoparticles may be a promising therapeutic platform for future applications in cancer molecular imaging and therapy.

However, it was demonstrated that unmodified TEVs are unlikely to be useful as a systemically administered tumor-specific delivery system. The rapid clearance of unmodified TEVs inhibits their accumulation in tumor tissue to any significant level, limiting their use as a drug delivery vehicle when injected intravenously (Smyth et al., 2015). More importantly, TEVs are well documented for their role in favoring cancer progression through enhanced cell proliferation and escape to apoptosis, induced angiogenesis, metabolic reprograming, boosted invasive, and disseminate ability, and escape from immune surveillance (Meng et al., 2019). Hence, unlike exosomes from other sources, TEVs may be a double-edged sword when used to deliver therapeutic agents for cancer treatment. Full elucidation of the formation, secretion, and networking function of TEVs is urgently needed for the realization of this attractive and promising strategy for cancer therapy (Sun et al., 2018). More extensive *in vivo* studies with large sample sizes are needed to investigate the effectiveness and safety of TEVs serving as a DDS in the future (Sun et al., 2018).

#### 3.1.6. Others

In addition to the above-mentioned cells, there are other candidates for drug delivery vesicles. For example, EVs derived from red blood cells (RBCs) (Kuo et al., 2017; Zhang et al., 2019), T cells (Lu et al., 2018), and natural killer (NK) cells (Zhu et al., 2018) have been investigated for their potential in drug delivery. Since RBCs are the most abundant cell type (84% of all cells) in the body, they are easy to obtain and are available in blood banks. In addition, CD47 on RBC-derived EVs is capable of interacting with its receptor signal regulatory protein alpha (SIRPa) on macrophages to protect RBCs-derived EVs from clearance by initiating the ‘don’t eat me’ signal (Tian et al., 2014). Usman et al. (2018) have described a strategy to generate large-scale amounts of RBC-derived EVs for the delivery of RNA drugs, including antisense oligonucleotides, Cas9 mRNA, and guide RNAs. RNA delivery by RBC-derived EVs showed highly robust gene inhibition and CRISPR-Cas9 genome editing *in vitro* and vivo with no observable cytotoxicity (Usman et al., 2018).

NK cell-derived EVs were found to contain tumor necrosis factor-α and granzyme B, exerting cytotoxic effects on glioblastoma (Zhu et al., 2018) and melanoma cells (Zhu et al., 2017) *in vitro* and *in vivo* without significant side effects. Activated CD8+ T cells from healthy mice have been found to release cytotoxic EVs causing remarkable attenuation of tumor invasion and metastasis (Seo et al., 2018). We have recently showed that γδ T cell-derived EVs load with miR-138 had direct anti-tumor and indirect tumor immune promoting effects on oral squamous cell carcinoma (Li et al., 2019c).

Thus, EVs from different cell sources contain diverse contents, exert different functions (Zheng et al., 2019), and distribute variously (Wiklander et al., 2015). The source of EVs is one of the most important factors that determine the drug delivery efficient, which is needed to be taken into careful consideration in the future.

### 3.2. EV ‘cargos’

#### 3.2.1. Small molecules

As mentioned above, many kinds of chemotherapeutic drugs have been tested for delivering by EVs, such as curcumin (Zhuang et al., 2011), paclitaxel (Agrawal et al., 2017), and doxorubicin (Zhang et al., 2018b). It was showed that curcumin encapsulated in exosomes can achieve three times anti-inflammatory activity than being delivered directly (Zhuang et al., 2011). EVs derived from human lung cancer cells are used for systemic delivery of oncolytic virus or paclitaxel with increased anti-tumor effects (Garofalo et al., 2019). The packaging of doxorubicin by EVs was found to have remarkably increased biological activity, targeting efficiency, and anti-tumor effect compared with doxorubicin delivered by liposome (Zhang et al., 2018b).

#### 3.2.2. Nucleic acids

EVs are natural carriers of nucleic acids molecules and can be genetically engineered to deliver specific nucleic acid molecules such as siRNA (El-Andaloussi et al., 2012), miRNA (Li et al., 2019c), and gene editing system-CRISPER/Cas9 (Lin et al., 2018). SiRNA and miRNAs can target complementary mRNAs for degradation in a sequence-dependent manner, but the low bioavailability and inability to cross key biological barriers such as the BBB make them difficult to translate into clinical application. Fortunately, double membrane structure of EVs with naturally secretory process can be developed for RNAi delivery. Indeed, many lines of evidence have shown promising therapeutic effects of EV-based nucleic acid delivery in cancer treatment (van den Boorn et al., 2011; Didiot et al., 2016; Kamerkar et al., 2017). However, more efforts are needed to screen out an ideal EV donator and loading protocol.

#### 3.2.3. Protein

Both the inside and the surface of EVs contain large amounts of protein molecules that provide binding sites to ligands of recipient cells. Haney et al. (2015) have developed an EV-based antioxidant and catalase delivery system for the treatment of Parkinson’s disease. Hong et al. (2018) designed a PH20 hyaluronidase delivery nano-system based on EV, which is able to penetrate deeply into tumor foci via hyaluronan degradation, allowing tumor growth inhibition and increased T cell infiltration. There are many other studies that have shown protein or peptide within exosomes provides an intended purpose to targeted therapy. However, the packaging of active proteins in EVs remains challenging.

#### 3.2.4. Metal nanoparticles

EVs loaded with metal nanoparticles, such as gold nanoparticles and iron oxide nanoparticles, have been tested for imaging diagnosis. Bose et al. (2018) developed gold-iron oxide nanoparticle packaging system by EVs. They showed that these gold-iron oxide rich EVs achieved excellent T2 contrast MRI and resulted in efficient photothermal effect in 4T1 cells. Recently, Perets et al. (2019) developed a method for longitudinal and quantitative *in vivo* neuroimaging of exosomes based on the superior visualization abilities of classical X-ray computed tomography (CT), combined with gold nanoparticles as labeling agents. Upon administered intranasally, gold nanoparticle-labeled exosomes could be efficiently tracked by CT (Perets et al., 2019). EVs loaded with metal nanoparticles provide a potential alternative diagnostic methodology for various pathological conditions. However, the efficiency, specificity, and safety of metal nanoparticle-loaded EVs need to be addressed in the future.

## 4. Advantages of EV-based drug delivery system

### 4.1. Limited immunogenicity and cytotoxicity

The competitive advantage of EVs as DDS compared with synthetic drug delivery reagents is their innate limited immunogenicity and cytotoxicity (Armstrong & Stevens, 2018). Currently, synthetic lipid nanoparticles, i.e. liposomes, are the mainstay for nucleic acid and small molecule delivery (Johnsen et al., 2018). Doxil^®^, the first liposomal anticancer drug formulation was approved by the FDA in 1995 (Barenholz, 2012). Among many other similar drugs appeared recently, toxicity is one of the reasons for delayed clinical applications of liposomes (Palazzolo et al., 2018). Indeed, synthetic lipid nanoparticles always induce a toxic immune response *in vivo*, and they accumulate in the liver mostly and do not perform as well as expected (De Jong & Borm, 2008; Zolnik et al., 2010; Fernandez-Fernandez et al., 2011). In contrast to synthetic lipid nanoparticles, EVs, due to their endogenous origin and high biocompatibility, are negatively challenged by immune clearance. Kamerkar et al. (2017) utilized exosomes to deliver siRNA to target the KRAS mutant protein. Their results showed that intravenously injected siRNA-loaded exosomes inhibited pancreatic cancer in mice better than siRNA-loaded lipid nanoparticles, without any obvious immune response (Kamerkar et al., 2017). This nature of EVs shed light on future DDS, although EVs are also accumulated in liver, and other important organs, such as pancreas. Nevertheless, compared with other delivery systems, such as adenoviruses, lentiviruses, retroviruses, lipid transfection reagents, and lipid nanoparticles, EV-based DDS is well recognized to have limited immunogenicity and cytotoxicity (van der Meel et al., 2014; Liu et al., 2019; Yang et al., 2019).

### 4.2. Stability in circulation

EVs are benefited from endogenous biogenesis machinery which determines that they should be highly stable *in vivo* (Bell et al., 2016; Armstrong et al., 2017). However, it is reported that EVs have a limited half-life of 2–20 min (Wiklander et al., 2015). This is substantially shorter than that of liposomes which have a half-life of up to several days (Immordino et al., 2006). Moreover, the liposomes used for drug delivery are often PEGylated, which further increase the circulating time. PEGylation, likewise, has been reported to remarkably prolong the circulating time of EVs to more than 60 minutes (Kooijmans et al., 2016). Thus, even being PEGylated, EVs still have less stability than liposomes at present. A problem with PEGylation is that a highly accelerated clearance of PEGylated nanoparticles may happen at repeated dosing, due to IgM antibodies raised against the PEG decoration (Børresen et al., 2018).

In addition to PEGylation, several other modifications have been reported to enhance the stability of EVs. For example, EVs derived from APCs can express membrane-bound complement regulators CD55 and CD59 to enhance the stability *in vivo* (Clayton et al., 2003). EVs could stay in circulation for a quite long time even when exposed to harsh inflammatory environment (Armstrong et al., 2017; Kutova et al., 2019). In addition, a CD47-mediated protection of EVs from mononuclear phagocytic systems expressed ‘don’t eat me’ signal which increases the time of EV in circulation (Kamerkar et al., 2017). What is more, a large number of studies demonstrated that due to the small size (≤100 nm), exosomes can achieve a targeting effect for tumor tissue via enhanced EPR effect (Bell et al., 2016; Yang et al., 2016). Nevertheless, much effort is definitely needed to strengthen the stability of EVs in further studies.

### 4.3. Cell targeting properties

Different cells under different conditions determine the heterogeneity of EVs, and different cell-derived EVs may be home to specific tissues (Thery et al., 1999; Colombo et al., 2014). For instance, EVs derived from hypoxic tumor cells tend to be taken up by hypoxic tumor cells (Jung et al., 2018). Again, central nervous system-derived EVs can cross the BBB and serve as a unique DDS for specific neuron populations (Shi et al., 2019). In addition, EVs derived from microglia cells can target multiple sclerosis and chronic inflammatory diseases of the central nervous system (Casella et al., 2018).

To better meet the future application of EVs as a controllable DDS, researchers have tried to modify EVs with ligands that can specifically bind to targeted cells. So far, three methods have been tried to modify EVs: (1) binding of receptor–ligand; (2) binding of antibody–antigen; (3) binding of microenvironment specific molecules. An example of receptor–ligand manner was that bioengineered EVs can specifically bind to HER2/Neu by expressing designed ankyrin repeat proteins (DARPins) on the membrane surface (Limoni et al., 2019). Brain and muscle targeting was achieved by bioengineering a fusion protein of Lamp2b with neuron-specific rabies viral glycoprotein (RVG) peptide and muscle-specific peptide on the surface of EVs, respectively (Alvarez-Erviti et al., 2011). The second modification method was demonstrated by Cheng et al. (2018a) who engineered anti-CD3 and anti-EGFR on the surface of the exosomes allowing a cross-link of T cells and EGFR + cancer cells and eliciting potent antitumor immunity. The third way to endow EVs targetability was illustrated by Hong et al. (2018) who showed that hyaluronidase engineered exosomes could degrade tumor extracellular matrix and enhance the permeability of T cells and drugs in the tumor milieu. Additionally, a pH-sensitive fusion polypeptide and cationic lipid material was designed for combined anchoring on the surface of exosome membrane, resulting in an efficient cytosolic release of the exosome. In this scenario, cationic lipids act as a ‘glue’ to support cellular uptake of EVs (Nakase & Futaki, 2015).

Collectively, an innate targetability of EVs can be achieved by selecting specific EV donator. Acquired targetability of EVs could be obtained by several bioengineering methods.

## 5. Bottlenecks of EV-based drug delivery

### 5.1. Lack of standardized isolation and purification method

One of the bottlenecks in the clinical application of EVs as drug carriers is that there is no uniform standard for the separation of EVs (Li et al., 2017; Merchant et al., 2017). EVs are widely found in blood (Ruivo et al., 2017), saliva (Nonaka & Wong, 2017), urine (Street et al., 2017), and other biological fluids as carriers of cellular information (Ferguson & Nguyen, 2016). Effective extraction and separation of these EVs from different sources for the purpose of drug delivery remain challenging (Nonaka & Wong, 2017; Street et al., 2017). Hitherto, five separation methods have been developed for isolation of EVs: (1) ultracentrifugation-based isolation techniques; (2) size-based isolation techniques; (3) immunoaffinity capture-based techniques; (4) precipitation; (5) microfluidics-based isolation techniques (Li et al., 2017). According to a worldwide survey (Gardiner et al., 2016), differential ultracentrifugation remains the most commonly used EV separation and concentration technique. Various other techniques, such as density gradients, precipitation, filtration, size exclusion chromatography (SEC), and immunoisolation, were applied by 5–20% of respondents each. According to MISEV2018 (Théry et al., 2018) of EVs separation guidelines, different methods may be positioned on a recovery vs. specificity grid, ranging from low to high in each dimension. We summarized current isolation and purification methods of EVs in Table 1, in which techniques were classified to the following four categories: (1) high recovery and low specificity: methods include precipitation kits/polymer (PEG), low molecular weight cutoff centrifugal filters, and high speed ultracentrifugation without gradient; (2) intermediate recovery and intermediate specificity: methods include size-exclusion chromatography (Stranska et al., 2018) and high molecular weight centrifugal filters (Vergauwen et al., 2017); (3) low recovery and high specificity: methods include filtration combined with SEC (Théry et al., 2018), immunoaffinity capture-based techniques, and microfluidics-based isolation techniques; (4) high recovery and high specificity: (not yet developed). An ideal isolation technique should be selective, easy-to-use, economical, reproducible, high-yield, time-saving, and high-throughput. Apparently, none of currently applied method meets these ideal criteria. Although various methods can be used for separation of EVs, we need to consider the following question: which method should we choose? What is the detail of this process? What kind of interesting EVs can be obtained? Realistically, to achieve standardized isolation and purification method, there will be a long way to go.

**Table 1.** Summary of isolation and purification method of EVs.

Isolation method	Principle of isolation	Characteristic	Grade of isolation efficiency
Ultracentrifugation-based isolation techniques	Density, size, and shape based sequential separations of particulate constituents and solutes	Large sample capacity and yielding of large amounts of exosomes, but high equipment cost, cumbersome, long run time and high speed centrifugation may damage exosomes	Low recovery and high specificity
Size-based isolation techniques	Size difference between exosomes and other particulate constituents	Low equipment cost and fast but shear stress induced deterioration and exosomes loss due to attaching to the filter membranes	Intermediate recovery and intermediate specificity
Immunoaffinity capture- based techniques	Specific interaction between membrane-bound antigens (receptors) of exosomes and immobilized antibodies (ligands)	Suitable for the isolation of specific exosomes with high specificity, but high reagent cost, exosome tags need to be established, low sample capacity and low yields	Low recovery and high specificity
Precipitation	Altering the solubility or dispersibility or exosomes by the use of water-excluding polymers	Easy to use, no need for special equipment, high sample capacity, but low specificity and co-precipitation of other non-exosomal contaminants like proteins and polymeric materials	High recovery and low specificity
Microfluidics-based isolation techniques	A variety of properties of exosomes like immunoaffinity, size, and density	Fast, low cost, portable, easy automation and integration, high portability, but low in sample capacity and no isolation standard	Low recovery and high specificity

### 5.2. Limited drug loading efficiency

The second main challenge in applying EVs to targeted therapies is to achieve an efficient loading of therapeutic cargoes into EVs (Luan et al., 2017). Similar to liposomes, interested therapeutic cargoes can be loaded into EVs by different ways. However, the loading efficiency for EVs is relatively lower than that for liposomes (Vader et al., 2016). The reason might be that EVs themselves contain part of the contents of their parent cells during the formation, resulting in limited space for the loading of exogenous drugs into EVs. Thus, the loading of exogenous drugs into EVs is a huge barrier (Lai et al., 2013; van der Meel et al., 2014; Li et al., 2018). Fortunately, several drug loading methods have been developed, which can be divided into three main categories: (1) pre-loading methods; (2) post-loading methods; (3) other loading methods. Pre-loading methods, such as transfection (Akao et al., 2011; Ohno et al., 2013; Batrakova & Kim, 2015) and co-incubation (Pascucci et al., 2014; Lee et al., 2015), encapsulate the drugs into the parental cells and drugs are loaded into EVs along with their formation process. The post loading protocols, such as incubation (Sun et al., 2010), electroporation (Tian et al., 2014), sonication (Kim et al., 2016), extrusion (Batrakova & Kim, 2015), and freeze/thaw cycle (Haney et al., 2015), are methods that package drugs directly into the EVs. Other loading methods include engineered parental cell and microfluidic synthesis of biomimetic lipid nanoparticles. For example, Li et al. (2019d) reported an engineered EV for RNA delivery by constructing a fusion protein CD9-HUR which has ultra-high affinity for miRNA, achieving enhanced RNA loading efficiency in EVs. Biomimetic lipid nanoparticles are therapeutic platforms with intrinsic biological characteristics and good delivery capacity that can partially mimic the cell surface profiles of RBCs, white blood cells, platelets, and even cancer cells. Rao et al. (2017) reported an improved cancer diagnosis and therapy by RBC membrane-capped magnetic nanoparticles.

In some reports, several methods were used for loading drugs into the same EVs, and thus, the methods could be accurately compared. We looked for consistent results from different studies to get the high, medium, and low loading methods. Table 2 summarizes the drug loading technique of EVs. For example, Haney et al. (2015) found the loading amount of catalase into EVs was increased in the row: the incubation at RT < freeze/thaw cycle < sonication ≈ extrusion. Kim et al. (2016) concluded the amount of PTX loaded into exosomes was increasing as follows: incubation at RT < electroporation ≪ sonication. Fuhrmann et al. (2015) reported the amount of loading drugs of saponin or hypotonic dialysis was up to 11-fold higher compared with incubation, electroporation, and extrusion. Obviously, saponin and hypotonic dialysis loading were high loading efficiency.

**Table 2.** Summary of drug loading technique of EVs.

Classification	Loading method	Type of cargo	Characteristic	Loading efficiency
Pre-loading method	Transfection	miRNA (Ohno et al., 2013), siRNA (Steinman, 2012), protein (Limoni et al., 2019)	Widely used but uncontrollable in quantity of cargo loading	Low loading efficiency
	Co-incubation	Paclitaxel (Merchant et al., 2017) carboplatin and etoposide (Akao et al., 2011)	Easy to operate but drugs may be cytotoxic to cells	Low loading efficiency
	Activities	miRNA (Pascucci et al., 2014)	Easy to operate but only applicable to specific cells	Low loading efficient
Post-loading method	Co-incubation	Curcumin (Street et al., 2017) hsiRNA (Seo et al., 2018), porphyrins (Lee et al., 2015), catalase (Li et al., 2019c)	A simplest way but uncontrollable in quantity of cargo loading	Low loading efficiency
	Electroporation	SiRNA (Steinman, 2012), TMP (Lee et al., 2015), DOX (Kantoff et al., 2010)	Superior loading of siRNA over chemical transfection but disrupting integrity of exosomes	Medium loading efficiency
	Sonication	PTX (Cheng et al., 2017), catalase (Li et al., 2019c), small RNAs (Sun et al., 2010)	High loading efficiency but not efficient for hydrophobic drugs	High loading efficiency
	Extrusion	Porphyrins (Lee et al., 2015), catalase (Li et al., 2019c)	High drug loading efficiency but potential deformation of membrane	High loading efficiency
	Freeze/thaw cycle	Catalase (Li et al., 2019c), prepare hybrid exosomes (Li et al., 2019d)	Exosomes may aggregate and the drugs loading efficiency is low	Low loading efficiency
	Saponin-assisted loading	Catalase (Li et al., 2019c), hydrophilic molecules (Lee et al., 2015)	High drug loading efficiency but generates pores in exosomes hemolysis/toxicity concerns	High loading efficiency

The loading capacity of EVs seems to be affected on the loading methods and the hydrophobic of the drug, while the different chemical lipid composition of the EVs was also important. In addition, more efforts are needed to optimize current loading technologies and develop novel methods in future studies.

### 5.3. Insufficient clinical grade production

The translation of this nanosystems into clinics encounters a major challenge concerning a production method that assures not only high quality but also high quantity (Lamparski et al., 2002; Momen-Heravi et al., 2013). With different methods, researchers have made much effort to obtain GMP grade EVs. In early 2002, Lamparski et al. (2002) described a production, purification, and characterization method for GMP-grade EVs from antigen presenting cells as a viable vaccine for cancer. Recently, Pachler et al. (2017) developed a GMP standard protocol for human MEVs. More recently, a GMP-grade method for the large-scale preparation of EVs from human cardiac progenitor cells was described (Andriolo et al., 2018). Additionally, Mendt et al. (2018) reported a bioreactor-based, large-scale production protocol of clinical-grade exosomes employing GMP standards. An ideal GMP-grade EV production method requires sterile generation of exosomes with therapeutic payloads, sufficient amounts for clinical testing, without batch-to-batch variation leading to compromised efficacy (Mendt et al., 2018). Apparently, there is not yet a state-of-the-art method that meets the ideal criteria for the production of large-scale GMP-grade EVs, a context in which scalability, reproducibility, safety, potency, size distribution, surface charge, and purity of the resulting product represent crucial issues (Pachler et al., 2017). Moreover, the question of the cell source remains unclear. EVs might mimic parent cell features, the cell type source may impact targeting and biological properties of EVs. Since the ideal EVs donator as well as EVs cargo has not yet reached consensus. An ideal GMP-grade EVs production method should depend on the specific EVs donator with a given cargo being loaded in.

## 6. Discussion

EVs are of great interest and importance in drug delivery. The promising advantages of low immunogenicity, intrinsic cell targeting properties, and enhanced stability in circulation make them more and more attractive in targeted drug therapy (Liao et al., 2019; Piffoux et al., 2019). They have been revealed huge potentials in the therapy of various diseases including malignancies and neurology diseases (Bunggulawa et al., 2018; Khan et al., 2018). Indeed, there is much preclinical evidence showing that systemic use of EVs as DDS can achieve a targeted therapeutic effect on specific scenarios. Liposome-based DDSs is relatively well clarified on their tiny changes in size, surface charge, flexibility in circulation, and ability to cross barriers. However, the biogenesis as well as transportation and uptake mechanisms of EVs remain largely abstruse. Moreover, EVs from different origin contain diverse contents and exert different functions (Zheng et al., 2019). The complexity of biochemical properties of EVs leads to many additional concerns, such as standard isolation and purification method, drug loading efficiency, and clinical-grade production. The specific benefits of EV-based drug delivery depend on the precise therapeutic requirements, i.e. the chemical nature of the drug, the mode of loading, the targeting disease site, and the mechanism of action. These characteristics have important implications for drug loading efficiency, cell uptake, administration pathways, and potential side effects.

In short, although there are significant challenges and difficulties in the application of EV-based drug delivery, this endogenous vesicle shows great potential in the biomedical field as the next generation of nanomaterials for advanced drug delivery and treatment.
